# Reorientation-induced relaxation of free OH at the air/water interface revealed by ultrafast heterodyne-detected nonlinear spectroscopy

**DOI:** 10.1038/s41467-020-19143-8

**Published:** 2020-10-22

**Authors:** Ken-ichi Inoue, Mohammed Ahmed, Satoshi Nihonyanagi, Tahei Tahara

**Affiliations:** 1 Molecular Spectroscopy Laboratory, RIKEN, 2-1 Hirosawa, Wako, Saitama 351-0198 Japan; 2 Ultrafast Spectroscopy Research Team, RIKEN Center for Advanced Photonics (RAP), 2-1 Hirosawa, Wako, Saitama 351-0198 Japan; 3 Present Address: Department of Chemistry, Graduate School of Science, Tohoku University, Sendai, 980-8578 Japan

**Keywords:** Physical chemistry, Chemical physics

## Abstract

The uniqueness of water originates from its three-dimensional hydrogen-bond network, but this hydrogen-bond network is suddenly truncated at the interface and non-hydrogen-bonded OH (free OH) appears. Although this free OH is the most characteristic feature of interfacial water, the molecular-level understanding of its dynamic property is still limited due to the technical difficulty. We study ultrafast vibrational relaxation dynamics of the free OH at the air/water interface using time-resolved heterodyne-detected vibrational sum frequency generation (TR-HD-VSFG) spectroscopy. With the use of singular value decomposition (SVD) analysis, the vibrational relaxation (*T*_1_) times of the free OH at the neat H_2_O and isotopically-diluted water interfaces are determined to be 0.87 ± 0.06 ps (neat H_2_O), 0.84 ± 0.09 ps (H_2_O/HOD/D_2_O = 1/2/1), and 0.88 ± 0.16 ps (H_2_O/HOD/D_2_O = 1/8/16). The absence of the isotope effect on the *T*_1_ time indicates that the main mechanism of the vibrational relaxation of the free OH is reorientation of the topmost water molecules. The determined sub-picosecond *T*_1_ time also suggests that the free OH reorients diffusively without the switching of the hydrogen-bond partner by the topmost water molecule.

## Abstract (web summary)

Water’s hydrogen-bond network is truncated at hydrophobic interfaces and the dynamics of the resulting free OH groups is not well understood. The authors experimentally show that the main vibrational relaxation mechanism for free OH at the air-water interface is a diffusive molecular reorientation, rather than intramolecular energy transfer.

## Introduction

Water is ubiquitous in nature and has unique physicochemical properties essential for realizing life, which attracts much attention in a broad range of scientific fields. In bulk, water molecules form a three-dimensional hydrogen-bond network that dynamically changes on a sub-picosecond time scale^1–6^. In contrast, at the air/water interface, such a three-dimensional hydrogen-bond network is truncated on the atomic scale, which is expected to cause distinct structures and dynamics of interfacial water. However, the molecular-level understanding of the properties of interfacial water is still limited owing to the technical difficulty of interface-selective measurements.

Vibrational sum-frequency generation (VSFG) is interface-selective spectroscopy based on the second-order nonlinear optical process, and it is a powerful method to investigate aqueous interfaces^7–14^. One of the most notable observations with VSFG is a sharp OH stretch band at ∼3700 cm^−1^ at the air/water interface, which appears with a broad hydrogen-bonded OH stretch band peaked at ∼3450 cm^−1^^15^. Such a sharp high-frequency band is absent in either IR or Raman spectrum of liquid water, and it is characteristic of the air/water interface. In fact, this 3700-cm^−1^ band arises from the non-hydrogen-bonded OH (free OH) of topmost water molecules that have OH pointing out to the air. This free OH has been attracting much interest experimentally and theoretically^16–19^, and it has been found that the “free” OH exists not only at the air/water interface but also at other hydrophobic interfaces^20^. Indeed, the free OH is now considered a molecular-level indicator of the hydrophobicity that plays critical roles in various chemical/biological processes such as membrane functions, on-water catalytic reactions, etc^21,22^. Therefore, the elucidation of the dynamic property of the free OH is very important, as a cornerstone for understanding interfacial water.

The ultrafast vibrational dynamics of water at interfaces was initially studied by conventional homodyne-detected time-resolved (TR−) VSFG^23–25^, and the free OH dynamics at the air/water interface was investigated by Bonn and coworkers^26–28^. They examined the effect of isotopic dilution to discuss the mechanism of vibrational relaxation because the intramolecular energy transfer is expected to be decelerated in HOD species due to the very large mismatch between the OH and OD stretch frequencies^29^. Since the vibrational relaxation (*T*_1_) time of the excited free OH derived by their homodyne-detected TR-VSFG experiments looked significantly changed with isotope dilution, they concluded that intramolecular energy transfer is the major relaxation pathway of the vibrationally excited free OH^28^.

Although it appears that the previous studies using homodyne detection provide fair evidence for the major role of the energy transfer in the relaxation process of the free OH, it has recently been recognized that homodyne detection has crucial problems, in particular for TR-VSFG measurements^13,30^. Actually, they can only provide the temporal change of the square of the second-order susceptibility (|*χ*^(2)^|^2^). The time-resolved spectra are the difference between the spectra measured with and without pump pulse irradiation so that those measured with homodyne detection correspond to Δ|*χ*^(2)^(*t*)|^2^ (=*χ*^(2)^Δ*χ*^(2)*^ + *χ*^(2)*^Δ*χ*^(2)^ – |Δ*χ*^(2)^|^2^). Obviously, it is very difficult to extract adequate spectral and dynamical information from the data obtained by homodyne TR-VSFG experiments. In contrast to homodyne VSFG, heterodyne-detected (HD−) VSFG provides the spectra that directly represent second-order susceptibility^9,10^. Therefore, time-resolved HD-VSFG (TR-HD-VSFG) allows us to obtain the pump-induced change of the imaginary part of *χ*^(2)^ (ΔIm*χ*^(2)^), which can directly be compared to TR-IR and TR-Raman spectra for the bulk^13^.

In the present study, the ultrafast vibrational relaxation dynamics of the free OH at the air/water interface is studied with TR-HD-VSFG for neat H_2_O and isotopically diluted water. Contrary to the previous homodyne TR-VSFG studies, we found that the relaxation *T*_1_ time of the free OH determined by TR-HD-VSFG does not change with isotopic dilution. The absence of the isotopic dilution effect as well as other observations indicates that the predominant vibrational relaxation mechanism of the excited free OH is not the intramolecular energy transfer but the reorientation of the topmost water molecule that has the free OH.

## Results

### Steady-state spectra of the air/water interfaces

**Fig. 1:**
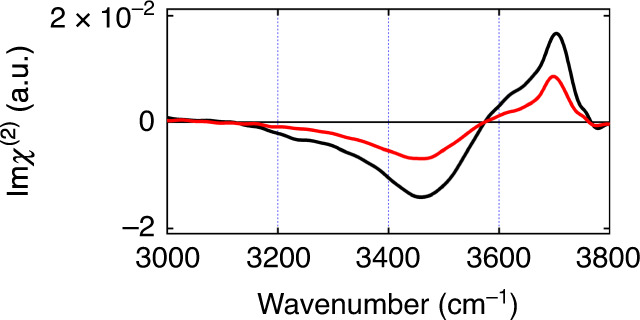
Steady-state Im*χ*^(2)^ spectra of the air/water interfaces. Steady-state Im*χ*^(2)^ spectra of the air/neat H_2_O interface (black) and the air/isotopically diluted water interface (red, H_2_O/HOD/D_2_O = 1/2/1).

Figure 1 shows the steady-state Im*χ*^(2)^ spectra of the air/neat H_2_O interface and the air/isotopically diluted water interface (H_2_O/HOD/D_2_O = 1/2/1). We adopted this isotopic dilution ratio for direct comparison with the previous homodyne VSFG study^28^. Both spectra exhibit a broad negative OH stretch band around 3150–3550 cm^−1^ and a sharp positive OH stretch band at 3700 cm^−1^. The former low-frequency broad band is assigned to the hydrogen-bonded OH (HB OH) of interfacial water, and its frequency and bandwidth are very similar to those of the HB OH band in the bulk IR spectrum. The latter high-frequency band is due to the free OH^31^, which we address in this study. The Im*χ*^(2)^ spectrum of the isotopically diluted water interface is similar to that of the H_2_O interface, except that the amplitude is about half due to the dilution. Time-resolved measurements are carried out by probing the whole OH stretch region with selective excitation of the free OH band.

### Time-resolved spectra measured with the free OH excitation

**Fig. 2:**
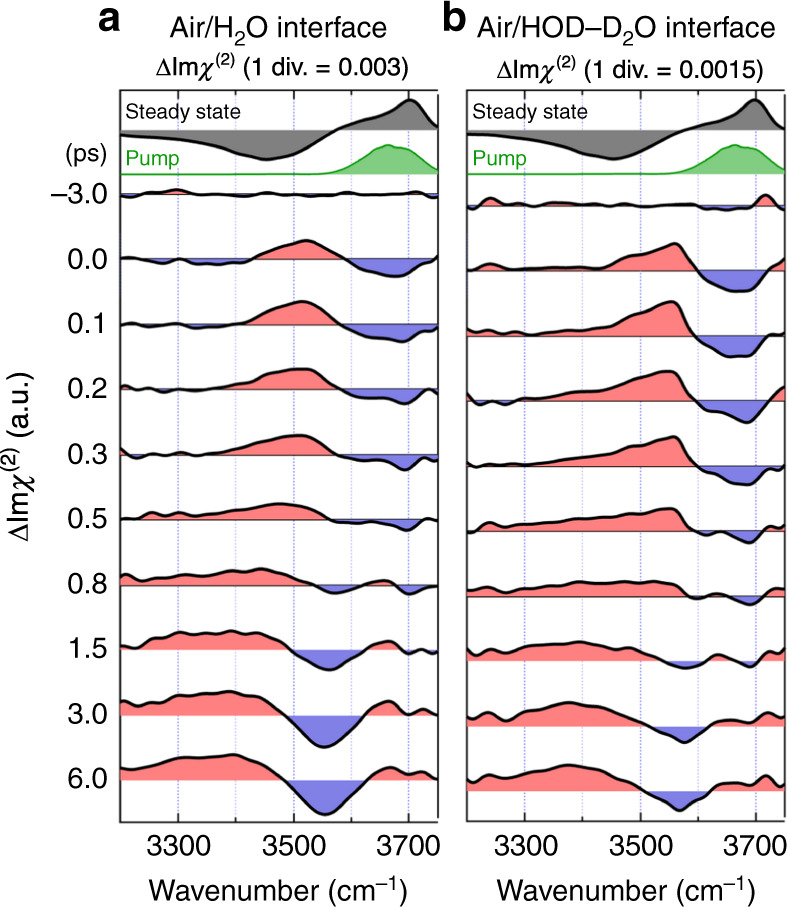
Time-resolved ΔIm*χ*^(2)^ spectra of the air/water interfaces. Time-resolved ΔIm*χ*^(2)^ spectra from −3.0 to 6.0 ps obtained with selective excitation of the free OH, **a** neat H_2_O and **b** isotopically diluted water (H_2_O/HOD/D_2_O = 1/2/1) interfaces. The time shown along the vertical axis represents the delay time of each time-resolved ΔIm*χ*^(2)^ spectrum. Red and blue regions in the spectra are positive and negative changes from the steady-state Im*χ*^(2)^ spectrum, respectively. The steady-state Im*χ*^(2)^ spectrum and the spectrum of the pump pulse are also shown at the top, for comparison.

Figure 2a shows the time-resolved ΔIm*χ*^(2)^ spectra of the air/neat H_2_O interface obtained with the free OH excitation at the delay times from −3.0 to 6.0 ps. The bandwidth of the pump pulse is ~100 cm^−1^, which enables selective excitation of the free OH. At −3.0 ps without pump influence, no transient signal is observed. At 0.0 ps, a negative signal at ∼3700 cm^−1^, a positive signal at ∼3500 cm^−1^, and a small negative signal below ∼3400 cm^−1^ are observed. The negative signal at ∼3700 cm^−1^ is readily assignable to the bleach of the fundamental transition (*v* = 1 ← *v* = 0) of the free OH because we selectively excite the free OH. This bleach band is accompanied by a positive band due to the excited-state transition (*v* = 2 ← *v* = 1), i.e., the hot band, which is seen at ∼3500 cm^−1^^32–34^. The small broad negative transient signal below 3400 cm^−1^ is attributable to the frequency shift of the HB OH band due to the anharmonic coupling between the HB OH and the free OH^30,35,36^. (This negative transient signal is more clearly seen in the expanded spectra shown in Supplementary Fig. 1). In other words, when the free OH of the topmost interfacial water molecule is excited, the intramolecular anharmonic coupling induces a redshift of the stretching frequency of another (HB−) OH in the same molecule. We note that this redshift of the negative HB OH band is expected to not only generate a negative feature in its low-frequency side but also give rise to a positive feature in its high-frequency side^36^. Therefore, the positive transient band around 3500 cm^−1^ should also have some contribution from this anharmonic frequency shift of the HB OH band. As the delay time increases, positive and negative signals appear at <3500 cm^−1^ and >3500 cm^−1^ in the HB OH region, respectively, reflecting a blue-shift of the HB OH band due to the temperature increase caused by the thermalization process^30,37^. This thermalized signal dominates the time-resolved spectra at later delay times.

The time-resolved ΔIm*χ*^(2)^ spectra of the air/isotopically diluted water interface (H_2_O/HOD/D_2_O = 1/2/1) are shown in Fig. 2b. Basically, the temporal change of the ΔIm*χ*^(2)^ spectra of the air/isotopically diluted water interface is very similar to that observed for the air/neat H_2_O interface: the ground-state bleach and the hot band of the free OH are observed in the early delay times, whereas the thermalized spectrum gradually dominates the ΔIm*χ*^(2)^ spectra at the later delay times. However, the negative signal below ∼3400 cm^−1^ at 0.0 ps is not clearly recognized in the spectrum of the air/isotopically diluted water interface. (The difference from the ΔIm*χ*^(2)^ at the air/neat H_2_O interface is more clearly recognized in the expanded spectra shown in Supplementary Fig. 1). This confirms the assignment of the weak negative signal observed at the air/neat H_2_O interface to the frequency shift due to anharmonic coupling because only H_2_O molecules having two OH’s can have the anharmonic coupling between the HB OH and the free OH. Because isotopically diluted water contains only 25% of H_2_O compared to the neat H_2_O, the weak negative signal arising from the anharmonic redshift of the HB OH band of H_2_O becomes much weaker, so that it cannot be recognized clearly with the signal to noise ratio of the present measurements. Thus, the absence of the 3400-cm^−1^ band at the isotopically diluted water interface strongly supports the assignment of the broad negative band at the neat H_2_O interface to the frequency shift due to the anharmonic coupling and warrants the high reliability of the obtained ΔIm*χ*^(2)^ spectra as well.

### *T*_1_ time of the free OH determined with SVD analysis

**Fig. 3:**
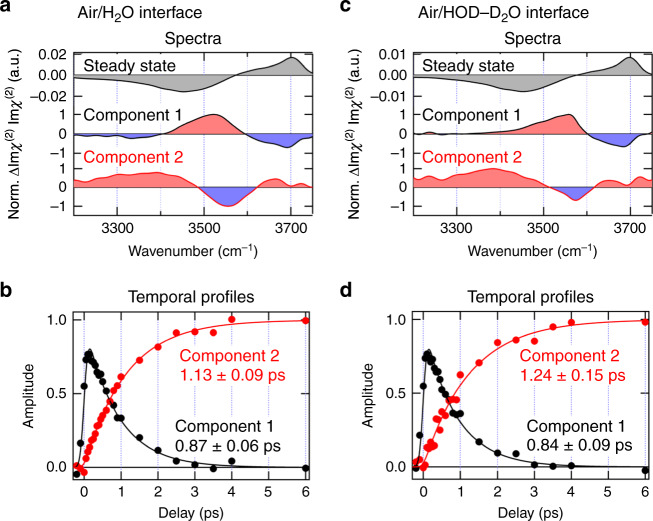
Singular value decomposition (SVD) analysis of the time-resolved ΔIm*χ*^(2)^ spectra. **a**, **c** The spectra and **b**, **d** the temporal profiles of the two major components obtained from SVD analysis of the time-resolved ΔIm*χ*^(2)^ spectra from −0.2 ps to 6.0 ps: **a**, **b** neat H_2_O and **c**, **d** isotopically diluted water (H_2_O/HOD/D_2_O = 1/2/1). Red and blue regions in the spectra (**a**, **c**) correspond to the positive and negative changes from the steady-state Im*χ*^(2)^ spectrum, respectively. The solid lines in **b**, **d** show the exponential fits for the decay and rise of the components, taking account of the instrumental response (200-fs Gaussian).

In order to extract quantitative information about the vibrational relaxation dynamics of the free OH by separating the thermalization signal, we carried out a singular value decomposition (SVD) analysis of the time-resolved ΔIm*χ*^(2)^ spectra obtained at the delay times from −0.2 to 6.0 ps. SVD is a mathematical technique to decompose a set of the time-resolved ΔIm*χ*^(2)^ spectra into independent spectral components and their temporal profiles (see Supplementary Note 2 for details)^38–40^. We stress that the SVD analysis is not applicable to time-resolved Δ|*χ*^(2)^|^2^ spectra measured with conventional homodyne detection and that it can be used for the time-resolved ΔIm*χ*^(2)^ spectra obtained with heterodyne detection. This is because the SVD analysis is only applicable to the spectra that are simple linear combinations of different spectral components. As shown in Fig. 3a, c, SVD successfully decomposes the ΔIm*χ*^(2)^ spectra into two spectral components. Component 1 represents the spectrum that is directly induced by the excitation of the free OH, whereas component 2 represents the spectrum after thermalization. The temporal profiles corresponding to these two components are shown in Fig. 3b, d. (Note that we assumed the exponential function for the temporal profiles of each component after SVD but the time constants themselves were determined by the analysis.) The most important result of this analysis is that the decay time constant of component 1, which corresponds to the *T*_1_ time of the free OH, is almost the same for the neat H_2_O (0.87 ± 0.06 ps) and the isotopically diluted water (0.84 ± 0.09 ps). This finding is very important for considering the relaxation mechanism of the free OH and is discussed in more detail later. The analysis also indicates a small difference between the *T*_1_ time of the free OH and the rise time of the thermalized spectrum. (1.13 ± 0.09 ps: neat H_2_O, 1.24 ± 0.15 ps: isotopically diluted water) This difference suggests the involvement of intermediate vibrational states (e.g., lower energy vibrational states) in the energy dissipation process of the excited free OH at the water interface.

As already mentioned, we chose the isotopic dilution ratio of H_2_O/HOD/D_2_O = 1/2/1 for direct comparison with the results of the previous homodyne TR-VSFG study^28^. Nevertheless, to confirm no detectable isotopic effect on *T*_1_, we also carried out time-resolved HD-VSFG experiments using isotopically diluted water with a higher dilution radio (H_2_O/HOD/D_2_O = 1/8/16). Although the signal to noise ratio of the obtained ΔIm*χ*^(2)^ spectra was substantially lower, the SVD analysis showed that the *T*_1_ time of the free OH (0.88 ± 0.16 ps) is the same as *T*_1_’s obtained for neat H_2_O and isotopically diluted water of H_2_O/HOD/D_2_O = 1/2/1 within the error (see Supplementary Note 3 for details).

### Vibrational relaxation mechanism of the free OH

**Fig. 4:**
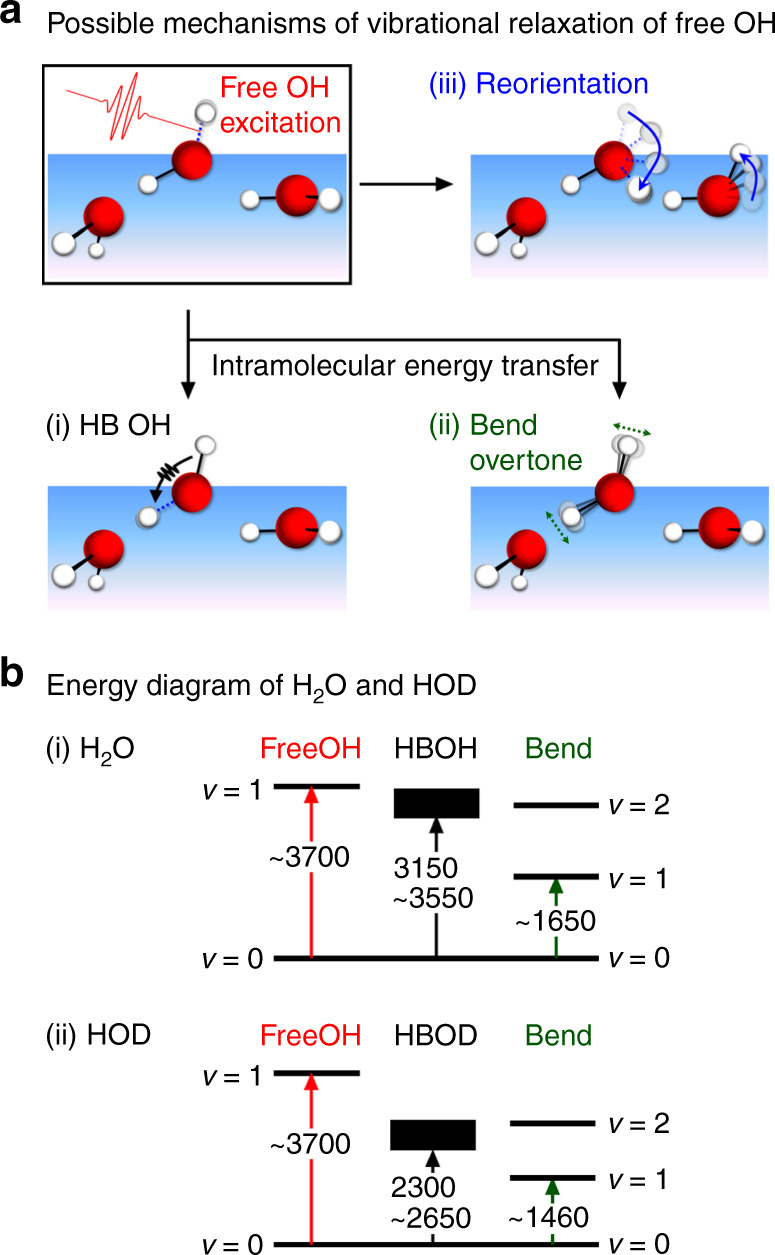
Possible relaxation mechanisms of the free OH and vibrational energy diagrams of interfacial water. **a** Three possible relaxation pathways for the vibrationally excited free OH. Intramolecular energy transfer from the excited free OH to (i) HB OH and (ii) bend overtone, and (iii) reorientation of the topmost water molecule. Arrows in (i), (ii), and (iii) depict the energy transfer, the bend vibration, and reorientational motion, respectively. Dotted lines represent the vibration in the excited state. **b** The vibrational energy levels of (i) H_2_O and (ii) HOD. The bending frequencies of H_2_O and HOD are the values of bulk water^42,43^.

There are three possible relaxation pathways for the vibrationally excited free OH^28^, which are depicted in Fig. 4a, i.e., (i) intramolecular energy transfer to the HB OH, (ii) intramolecular energy transfer to the bend overtone, and (iii) reorientation of the interfacial water molecules that change the free OH to the HB OH. In the case of (iii) the reorientation mechanism, while an excited free OH is relaxed with reorientation, an unexcited HB OH or an unexcited free OH that lies nearly horizontal becomes a new unexcited free OH, recovering the population of the free OH in the vibrationally ground state. Because the reorientation time of an OH group in HOD is considered essentially the same as that of an OH group in H_2_O, the relaxation time with this mechanism is not affected by the isotopic dilution. In the other two relaxation mechanisms, i.e., the intramolecular energy transfer from the free OH to HB OH stretch and that to the bend overtone, the relaxation is expected to be strongly decelerated in HOD generated with isotopic dilution, because of a large frequency mismatch between the OH and OD stretch vibrations and that between the OH stretch and the HOD bend overtone (Fig. 4b)^41–43^. The present experiment shows that the *T*_1_ time of the excited free OH does not change with the isotopic dilution. This absence of the isotope effect negates the primary role of the intramolecular energy transfer in the vibrational relaxation of the excited free OH. The present TR-HD-VSFG measurements strongly indicate that the reorientation of the topmost water molecules is the main relaxation mechanism of the free OH present at the air/water interface. In other words, the *T*_1_ time of the free OH is directly related to its reorientation time^26^.

### HB OH region: comparison with homodyne detection

To justify the above conclusion derived from the isotope effect on the *T*_1_ time of the free OH, we also examine the rise of the transient signal in the HB OH region. Actually, in the previous homodyne-detected TR-VSFG studies^27,28^, the intensity change in the HB OH region was measured with excitation of the free OH, and it was argued that the rise of the transient signal was significantly delayed compared to the appearance of the transient in the free OH region. Then, this delayed rise was considered as evidence of the intramolecular energy transfer from the free OH to the HB OH. In our assignment, however, the transient signal in the HB OH region is due to the hot band of the free OH and the anharmonic coupling between the free OH and HB OH. Therefore, if our assignment is correct, the transient signal in the HB OH region is expected to appear simultaneously with the bleaching signal in the free OH region.

**Fig. 5:**
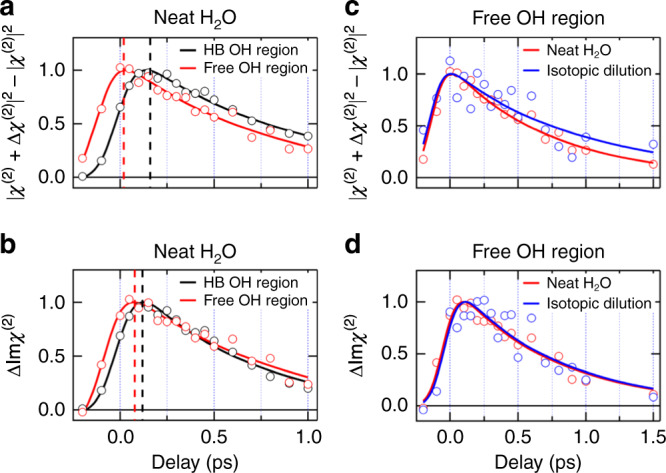
Comparison between the temporal traces of the time-resolved VSFG signals obtained with homodyne and heterodyne detections. **a** Homodyne TR-VSFG signals and **b** time-resolved ΔIm*χ*^(2)^ in the HB OH region (3500 cm^−1^, black) and the free OH region (3700 cm^−1^, red) at the air/neat H_2_O interface. Black and red dotted lines represent the peak of each temporal trace. **c** Homodyne TR-VSFG signals and **d** time-resolved ΔIm*χ*^(2)^ in the free OH region at the air/neat H_2_O interface (red) and isotopically diluted water interface (blue). Homodyne TR-VSFG signals in **a** and **c** are calculated from the data obtained in the present TR-HD-VSFG measurements as |*χ*^(2)^ + Δ*χ*^(2)^|^2^ – |*χ*^(2)^|^2^. Solid lines are the exponential fits convoluted by the instrumental response (200-fs Gaussian).

Figure 5a, c shows |*χ*^(2)^ + Δ*χ*^(2)^|^2^ – |*χ*^(2)^|^2^ calculated from the data obtained by the present TR-HD-VSFG measurements, which correspond to the signal obtained with the homodyne TR-VSFG measurement. (Time-resolved spectra of the real part of *χ*^(2)^, ΔRe*χ*^(2)^, used for the calculation are shown in Supplementary Fig. 4). As clearly seen in Fig. 5a, the rise of the |*χ*^(2)^ + Δ*χ*^(2)^|^2^ – |*χ*^(2)^|^2^ signal in the HB OH region (3500 cm^−1^) is substantially delayed compared to that in the free OH region, which reproduces the observation in the previous homodyne-detected TR-VSFG study^27,28^. However, the temporal change of the time-resolved ΔIm*χ*^(2)^ signal in the HB OH and free OH regions is very similar to each other (Fig. 5b). This clearly demonstrates that the large delay reported for the rise of the transient signal in the HB OH region predominantly arises from the artifact due to the homodyne detection, not due to the intramolecular energy transfer. We also note that a significant isotope effect is seen for the decay of the |*χ*^(2)^ + Δ*χ*^(2)^|^2^ – |*χ*^(2)^|^2^ signal in the free OH region (Fig. 5c: 0.75 ± 0.04 ps (H_2_O) and 1.34 ± 0.55 ps (HOD)) as reported, whereas it is absent in the ΔIm*χ*^(2)^ signal (Fig. 5d: 0.74 ± 0.06 ps (H_2_O) and 0.76 ± 0.29 ps (HOD)). This demonstrates that the reported isotopic dilution effect on the decay of the transient signal in the free OH region is also due to the artifact originating from the homodyne detection. It is noted that the present analysis on |*χ*^(2)^ + Δ*χ*^(2)^|^2^ – |*χ*^(2)^|^2^ and the previous homodyne TR-VSFG study^28^ look to provide somewhat different decay time constants for the transient signal in the free OH region. The reason for this discrepancy is not clear at the moment, but it may be attributable to the difference in the excitation condition such as the pump pulse energy and the focus size. Indeed, the temporal change of |*χ*^(2)^ + Δ*χ*^(2)^|^2^ – |*χ*^(2)^|^2^ is changed with the relative magnitude between *χ*^(2)^ and Δ*χ*^(2)^, and Δ*χ*^(2)^ strongly depends on the excitation condition.

Although the large delay of the rise of the transient signal in the HB OH region is predominantly attributed to the artifact due to homodyne detection, there still exists a small (∼50 fs) difference between the rises of transient signals in the free OH and HB OH regions (Fig. 5b). This 50-fs difference observed in the time-resolved ΔIm*χ*^(2)^ signal is attributable to the effect of the perturbed free induction decay (PFID)^44–46^. It is known that when the pump pulse arrives before the complete dephasing of a probed vibration, the pump perturbs the free induction decay of the vibration and gives rise to transient signals in the negative delay time region. Indeed, the PFID is clearly recognized in the time-domain signals of the SFG electric field which are obtained with Fourier transformation. (See Supplementary Note 5 for the details.)

The time-resolved ΔIm*χ*^(2)^ data obtained in this study clearly show that the temporal evolution of the pump-induced changes in the HB OH region is essentially the same as that in the free OH region, except for the effect of PFID. This means that the transient signal in the HB OH region reflects the free OH dynamics and that it arises from the hot band of the free OH and the spectral shift due to the anharmonic coupling. Thus, the analysis of the transient signal in the HB OH region also supports that the excited free OH predominantly relaxes by the reorientation mechanism, not through intramolecular energy transfer. Actually, this conclusion is readily rationalized by the almost no overlap between the vibrational frequencies of the free OH and HB OH, which makes resonant energy transfer inefficient. The energetic isolation of the free OH also explains why the relaxation time of the excited free OH is substantially longer than the *T*_1_ time of the HB OH (0.29–0.35 ps in bulk^6,47,48^ and ∼0.4 ps at the neat H_2_O interface^30^).

## Discussion

Lastly, we discuss the reorientation mechanism of the topmost water molecule based on the sub-picosecond relaxation time determined in this study. In bulk, the reorientation mechanism of water molecules has been discussed in the past decades, and it is now widely accepted that water molecules reorient with the jump mechanism in which hydrogen bonds are broken and reformed simultaneously in the reorientation process^5,49^. Polarization anisotropy decay measurements determined that this process proceeds with 2.5 ps^50,51^. At the interface, on the other hand, several theoretical studies suggest that the free OH reorients in a diffusive manner without switching the hydrogen-bond partner of the relevant topmost water molecule and that it proceeds faster than the reorientation by the jump mechanism^52,53^. The sub-picosecond relaxation time determined in this study accords with this theoretical prediction about the diffusive nature of the reorientation process of the topmost water molecules at the interface.

It may be worth noting that the picture of the reorientation-induced relaxation of the free OH obtained by the present TR-HD-VSFG study is in the same line with the conclusion of a recent homodyne-detected TR-VSFG study that reported the relaxation dynamics of the free OH at the water interface with the hydrophobic octadecylsilane self-assembled monolayer on fused silica^54^. Although experiments with heterodyne detection are desired also for such buried silica/monolayer interfaces, the similar conclusions for the two prototypical hydrophobic interfaces suggest the general importance of the reorientation relaxation pathway in the vibrational relaxation of the free OH at hydrophobic interfaces.

**Fig. 6:**
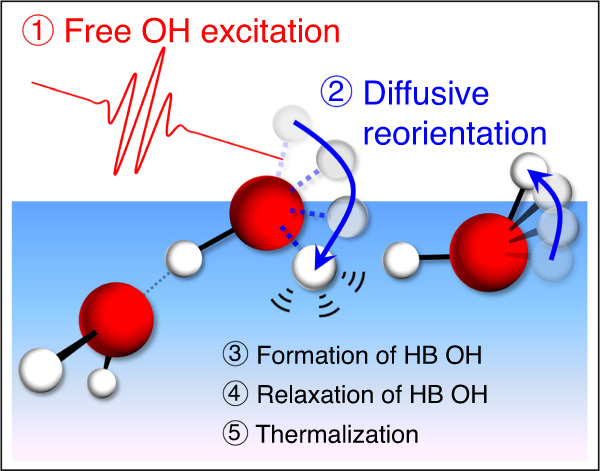
Schematic of the vibrational relaxation mechanism of the excited free OH. When a free OH is vibrationally excited (process 1), the excited free OH diffusively reorients (process 2) to form an excited-state HB OH (process 3). The excited HB OH rapidly relaxes to the lower energy vibrational states (process 4). Finally, the pump energy absorbed by water converts to the thermal energy (process 5). Dotted OH bonds represent the vibration in the excited state.

In summary, the ultrafast vibrational relaxation dynamics of the free OH at the air/water interface was studied using TR-HD-VSFG with neat H_2_O and isotopically diluted water. Using SVD analysis, we determined the *T*_1_ time of the excited free OH from the time-resolved ΔIm*χ*^(2)^ spectra, which are 0.87 ± 0.06 ps at the neat H_2_O interface, and 0.84 ± 0.09 ps (H_2_O/HOD/D_2_O = 1/2/1), and 0.88 ± 0.16 ps (H_2_O/HOD/D_2_O = 1/8/16) at the isotopically diluted water interfaces. The absence of the isotope effect on the *T*_1_ time strongly indicates that the vibrational relaxation of the excited free OH predominantly proceeds with the reorientation of interfacial water molecules, not through the intramolecular energy transfer. This means that the free OH is energetically isolated from the other vibrational modes. In addition, the sub-picosecond time constant of this reorientation-induced vibrational relaxation supports the diffusive nature of the free OH reorientation. Consequently, the picture of the vibrational relaxation process of the free OH obtained by this study is described as follows: When a free OH is vibrationally excited, the excited free OH is changed into an excited-state HB OH with diffusive reorientation. This process causes the loss of an excited free OH, which is compensated by the appearance of an unexcited free OH generated from an unexcited HB OH or an unexcited free OH lying almost horizontally, resulting in the recovery of the bleach of the unexcited free OH signal. The excited HB OH created by the reorientation of the free OH rapidly relaxes to the lower energy vibrational states as in the case of direct excitation of HB OH^30^. Finally, the pump energy absorbed by water converts to the thermal energy, which is observed as the appearance of the thermalized spectrum. A small difference between the *T*_1_ time of the free OH and the rise time of the thermalized spectrum suggests the involvement of other vibrational states (e.g., low-lying vibrational states) in this energy dissipation process at the water interface. The picture obtained in this study is sketched in Fig. 6. The air/water interface is the simplest and most fundamental hydrophobic interface. Therefore, the vibrational relaxation mechanism of the free OH at the air/water interface, which has been clarified in the present study, provides an indispensable basis for understanding the energy transport process at the hydrophobic interfaces.

## Methods

### Time-resolved heterodyne-detected vibrational sum-frequency generation (TR-HD-VSFG) spectroscopy

The optical setup for TR-HD-VSFG measurements was described in detail previously^55^. Briefly, a narrow-band visible *ω*_1_ pulse (center wavelength: 795 nm, bandwidth: 25 cm^−1^, pulse width: 0.5 ps, *s*-polarized) and a broadband infrared *ω*_2_ pulse (center frequency: 3450 cm^−1^, bandwidth: 300 cm^−1^, pulse width: 0.1 ps, *p*-polarized) were focused into a y-cut quartz crystal and then onto the air/water interface to generate sum frequency (*ω*_1_ + *ω*_2_, *s*-polarized). The former SFG was used as the local oscillator (LO) and passed through a glass plate (2 mm) in advance to be delayed by 3.2 ps with respect to the latter SFG from the sample. The spectral interferograms were recorded by using a polychromator with a charge-coupled device (CCD). The time-resolved measurements were carried out with pump *ω*_pump_ pulses at ∼3700 cm^−1^ (bandwidth: 100 cm^−1^, pulse width: 0.2 ps, *p*-polarized, 30 μJ) for selective excitation of the free OH. The time-resolution of the measurements was evaluated by measuring the third-order nonlinear signal of *ω*_1_ + *ω*_2_ + *ω*_pump_ that was generated with the z-cut quartz placed at the sample position while changing the *ω*_pump_ delay. The FWHM of the temporal response was ∼200 fs. The signals obtained with a 20-second exposure were accumulated 380 times and 560 times for the neat H_2_O and isotopically diluted water (H_2_O/HOD/D_2_O = 1/2/1), respectively, at each delay time.

## Supplementary information

Supplementary Information

## Data Availability

The data that support the findings of this study are available from the corresponding author upon reasonable request.
