# Supplementary material for: Reorientation-induced relaxation of free OH at the air/water interface revealed by ultrafast heterodyne-detected nonlinear spectroscopy
Source: Nat Commun. 2020 Oct 22;11:5344. doi: 10.1038/s41467-020-19143-8 (PMC7581742; doi:10.1038/s41467-020-19143-8)
Supplement: Supplementary file 1 — Supplementary Information [file 41467_2020_19143_MOESM1_ESM.pdf]

Supplementary Information for:

Reorientation-induced relaxation of free OH at the air/water interface revealed by ultrafast heterodyne-detected nonlinear spectroscopy

Ken-ichi Inoue,[1,#] Mohammed Ahmed,[1,2] Satoshi Nihonyanagi,[1,2] and Tahei Tahara[1,2]*

[1] *Molecular Spectroscopy Laboratory, RIKEN, 2-1 Hirosawa, Wako, Saitama 351-0198, Japan*

[2] *Ultrafast Spectroscopy Research Team, RIKEN Center for Advanced Photonics (RAP), 2-1 Hirosawa, Wako, Saitama 351-0198, Japan*

[#] *Present address: Department of Chemistry, Graduate School of Science, Tohoku University, Sendai 980-8578, Japan.*

*Correspondence to: tahei@riken.jp

Supplementary Notes

1. **The time-resolved $\Delta\mathrm{Im}\chi^{(2)}$ spectra at the air/water interface at 0.0 ps**
2. **SVD analysis of the time-resolved $\Delta\mathrm{Im}\chi^{(2)}$ spectra at the air/water interface**
3. **T_1 time of the free OH of the air/isotopically-diluted water interface (H_2O/HOD/D_2O = 1/8/16)**
4. **Time-resolved $\Delta\mathrm{Re}\chi^{(2)}$ spectra of the air/water interfaces**
5. **Influence of the perturbed free induction decay (PFID)**

Supplementary Note 1: The time-resolved $\Delta \mathrm{Im}\chi^{(2)}$ spectra at the air/water interface at 0.0 ps

Supplementary Figure 1 shows the expanded view of the time-resolved $\Delta \mathrm{Im}\chi^{(2)}$ spectra of the air/H_2O and the air/isotopically-diluted water interfaces at −3.0 and 0.0 ps. The spectrum of the air/H_2O interface at 0.0 ps exhibits the negative signal below 3400 cm^{-1} beyond the S/N level (Supplementary Figure 1 (a)), whereas the spectrum of the air/isotopically-diluted water interface does not (Supplementary Figure 1 (b)). This spectral difference supports that the broad negative band below 3400 cm^{-1} at the neat H_2O interface arises from the frequency shift of the HB OH band due to anharmonic coupling.

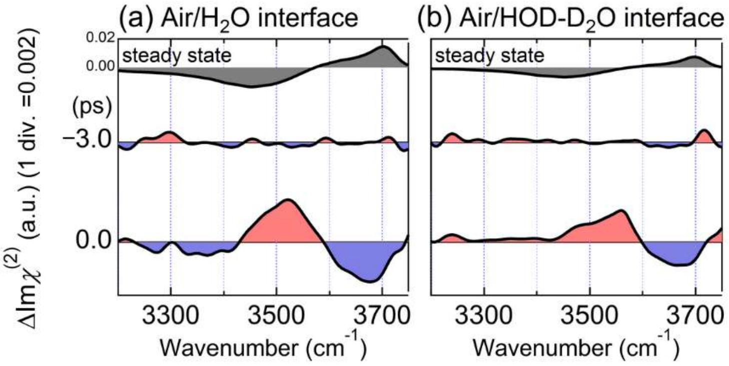

Supplementary Figure 1. **Expanded view of the time-resolved $\Delta \mathrm{Im}\chi^{(2)}$ spectra.** The time-resolved $\Delta \mathrm{Im}\chi^{(2)}$ spectra of (a) the air/H_2O and (b) the air/isotopically-diluted water (H_2O/HOD/D_2O = 1/2/1) interfaces at −3.0 ps and 0.0 ps in Figure 2 (a).

Supplementary Note 2: SVD analysis of the time-resolved $\Delta \mathrm{Im}\chi^{(2)}$ spectra at the air/water interface

We used the singular value decomposition (SVD) analysis to decompose the time-resolved $\Delta \mathrm{Im}\chi^{(2)}$ spectra.[1-3] The SVD analysis mathematically decomposes a set of time-resolved $\Delta \mathrm{Im}\chi^{(2)}$

spectra listed in matrix M into the product of two orthogonal matrices (U and V) and a diagonal matrix (W),

$$M = UWV^* = (\vec{u}_1 \quad \vec{u}_2 \quad \cdots)\begin{pmatrix} w_1 & 0 & \cdots \\ 0 & w_2 & \\ \vdots & & \ddots \end{pmatrix}\begin{pmatrix} \vec{v}_1^* \\ \vec{v}_2^* \\ \vdots \end{pmatrix}, \quad \text{(S1. 1)}$$

where, U, V, and W represent the spectrum, its temporal profile and the corresponding singular value of each component, respectively. The analysis showed that W has only two major diagonal elements (singular values), which are shown in Supplementary Figure 2 with the corresponding components in U and V. This implies that only two components (black and red in each figures) practically contribute to the time-resolved $\Delta\mathrm{Im}\chi^{(2)}$ spectra measured in this study. Thus, Eq. (S1. 1) can be simplified to Eq. (S1. 2):

$$M \simeq (\vec{u}_1 \quad \vec{u}_2)\begin{pmatrix} w_1 & 0 \\ 0 & w_2 \end{pmatrix}\begin{pmatrix} \vec{v}_1^* \\ \vec{v}_2^* \end{pmatrix}. \quad \text{(S1. 2)}$$

Eq. (S1. 2) implies that all the time-resolved $\Delta\mathrm{Im}\chi^{(2)}$ spectra are well reproduced by the linear combinations only of the two spectral components, $\vec{u}_1$ and $\vec{u}_2$. However, SVD is a purely mathematical procedure, and the spectral components ($\vec{u}_1, \vec{u}_2$) and their temporal profiles ($\vec{v}_1^*$, $\vec{v}_2^*$) obtained from the SVD analysis are orthogonalized, so that they do not have any physical meanings as they are. Therefore, for converting ($\vec{u}_1, \vec{u}_2$) and ($\vec{v}_1^*$, $\vec{v}_2^*$) to physically meaningful spectra and temporal profiles, we need to make their proper linear combinations with proper assumptions, as expressed with Eq. (S1. 3) using a transformation matrix (c_{ij}) ($i, j = 1, 2$).

$$M \simeq (\vec{u}_1 \quad \vec{u}_2)\begin{pmatrix} w_1 & 0 \\ 0 & w_2 \end{pmatrix}\begin{pmatrix} c_{11} & c_{12} \\ c_{21} & c_{22} \end{pmatrix}^{-1}\begin{pmatrix} c_{11} & c_{12} \\ c_{21} & c_{22} \end{pmatrix}\begin{pmatrix} \vec{v}_1^* \\ \vec{v}_2^* \end{pmatrix} = (\vec{u}_1' \quad \vec{u}_2')\begin{pmatrix} \vec{v}_1'^* \\ \vec{v}_2'^* \end{pmatrix}, \quad \text{(S1. 3)}$$

with

$$(\vec{u}_1' \quad \vec{u}_2') = \left(\frac{c_{22}w_1\vec{u}_1 - c_{21}w_2\vec{u}_2}{c_{11}c_{22} - c_{12}c_{21}} \quad \frac{-c_{12}w_1\vec{u}_1 + c_{11}w_2\vec{u}_2}{c_{11}c_{22} - c_{12}c_{21}}\right), \tag{S1. 4}$$

$$\begin{pmatrix} \vec{v}_1'^* \\ \vec{v}_2'^* \end{pmatrix} = \begin{pmatrix} c_{11}\vec{v}_1^* + c_{12}\vec{v}_2^* \\ c_{21}\vec{v}_1^* + c_{22}\vec{v}_2^* \end{pmatrix}. \tag{S1. 5}$$

In the present study, the coefficients c_{ij} were adjusted so that single exponential decay and rise (that are convoluted with 200-fs Gaussian instrumental response) were obtained for $\vec{v}_1'^*$ and $\vec{v}_2'^*$, respectively. With these procedures, we obtained the spectra and temporal profiles shown in Figure 3 in the main text.

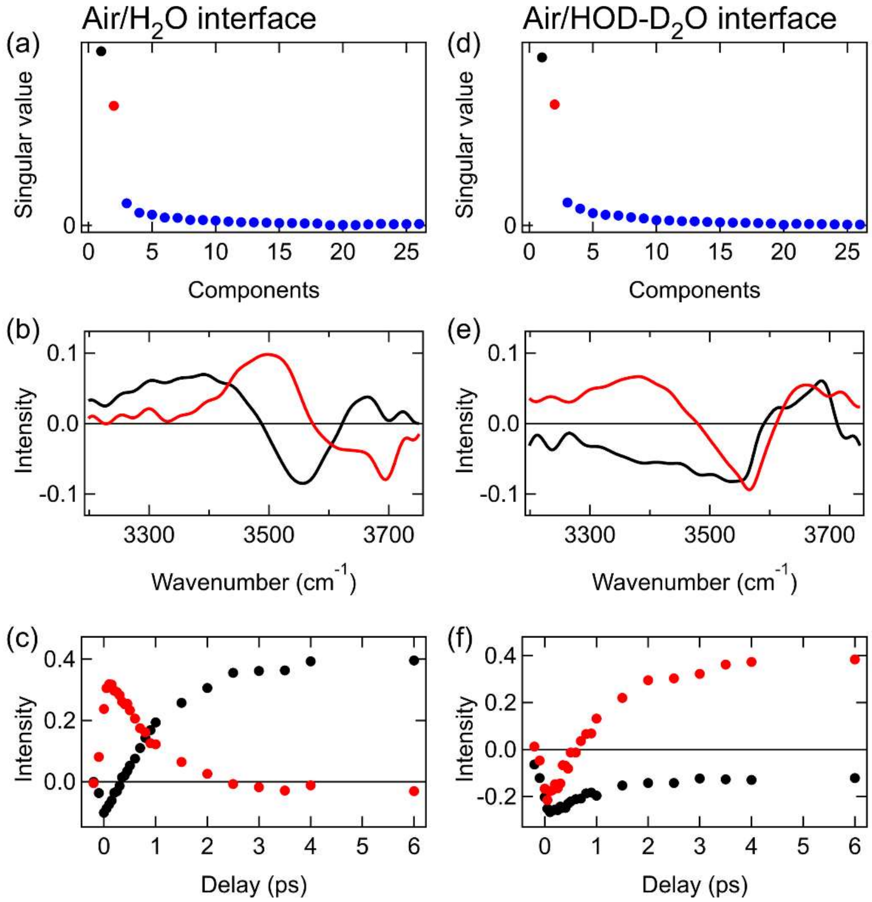

Supplementary Figure 2. **Direct results of the SVD analysis of the time-resolved $\Delta\mathrm{Im}\chi^{(2)}$ spectra.** (a) (d) Singular values, (b) (e) spectral components, and (c) (f) temporal profiles for

time-resolved $\Delta\text{Im}\chi^{(2)}$ data at delay times from −0.2 ps to 6.0 ps of the interface of neat H_2O ((a) (b) (c)) and isotopically-diluted water ($H_2O/HOD/D_2O$ = 1/2/1) ((d) (e) (f)). Components 1 and 2 at each interface are shown in black and red, respectively, in all figures. Singular values of other components are shown in blue in (a) and (d).

Supplementary Note 3: T_1 time of the free OH obtained with higher isotopic dilution ($H_2O/HOD/D_2O$ = 1/8/16)

In order to confirm the absence of the isotope effect on the T_1 time of the free OH, TR-HD-VSFG experiments were carried out also for the air/isotopically-diluted water interface prepared with a higher isotopic-dilution ratio ($H_2O/HOD/D_2O$ = 1/8/16). Supplementary Figure 3 (a) shows the time-resolved $\Delta\text{Im}\chi^{(2)}$ spectra obtained at the delay times from −3.0 ps to 6.0 ps. Basically, the temporal change of the $\Delta\text{Im}\chi^{(2)}$ spectra is very similar to those observed for the air/neat H_2O interface and air/isotopically-diluted water interface with a lower isotopic dilution ratio ($H_2O/HOD/D_2O$ = 1/2/1), which are shown in the main text: the ground-state bleach and the hot band of the free OH are observed in the early delay times, and the thermalized spectrum gradually dominates the $\Delta\text{Im}\chi^{(2)}$ spectra at the later delay times. Note that the signal-to-noise ratio of the $\Delta\text{Im}\chi^{(2)}$ spectra obtained with the higher isotopic dilution ratio ($H_2O/HOD/D_2O$ = 1/8/16) is much lower than those of the spectra shown in Figure 2 in the main text because of the much weaker signal intensity. Nevertheless, the SVD analysis successfully decomposed the time-resolved $\Delta\text{Im}\chi^{(2)}$ spectra into two major spectral components, and the spectrum and the relevant temporal profile of each component is shown in Supplementary Figure 3 (b) and (c), respectively. The component 1 represents the spectrum that is directly induced by the excitation of the free OH whereas the component 2 represents the spectrum after thermalization. The T_1

time of the free OH was obtained as 0.88 ± 0.16 ps, which is indistinguishable from the value obtained for the neat H_2O (0.87 ± 0.06 ps) and isotopically-diluted water with a lower isotopic dilution ratio (H_2O/HOD/D_2O = 1/2/1) (0.84 ± 0.09 ps) within the error. This result further confirms the absence of the isotope effect on the T_1 time of the excited free OH.

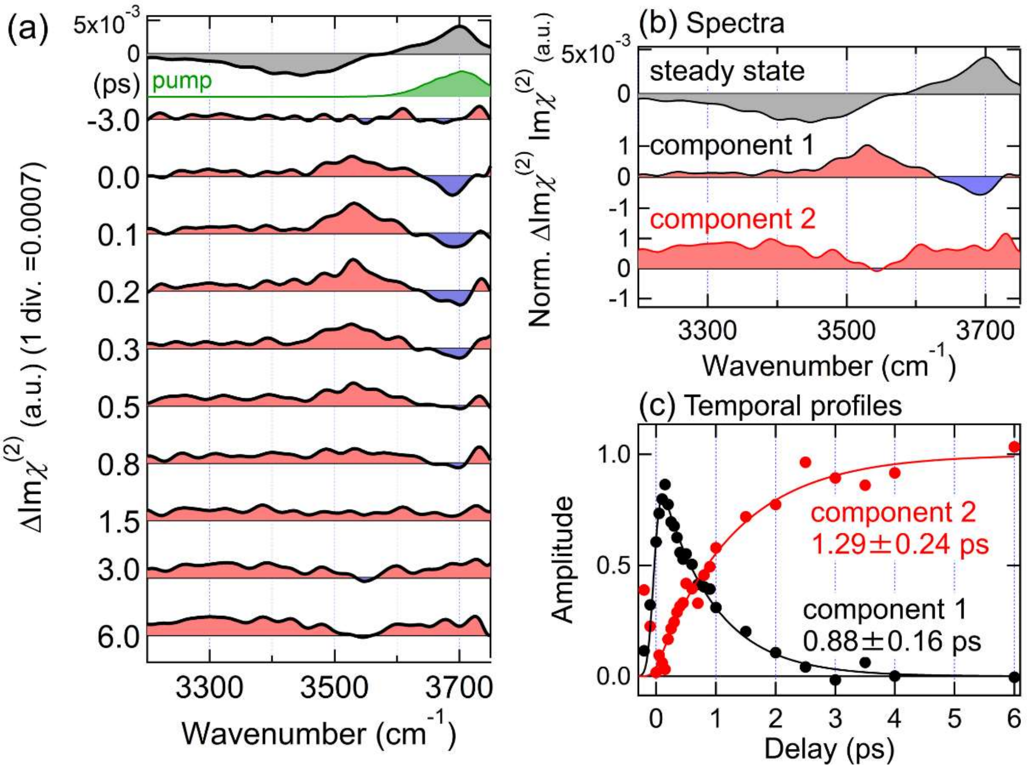

Supplementary Figure 3. **Vibrational dynamics of the air/isotopically-diluted water interface with a higher isotopic-dilution ratio (H_2O/HOD/D_2O = 1/8/16).** (a) Time-resolved $\Delta\mathrm{Im}\chi^{(2)}$ spectra obtained with selective excitation of the free OH. Steady-state $\mathrm{Im}\chi^{(2)}$ spectrum and the spectrum of the pump pulse are also shown at the top, for comparison. (b) The spectra and (c) the temporal profiles of the two major components obtained from the SVD analysis of the time-resolved $\Delta\mathrm{Im}\chi^{(2)}$ spectra at the delay times from –0.2 ps to 6.0 ps. The solid lines in (c) show the exponential fits to the decay and rise of the relevant components, which takes account of the instrumental response (200-fs Gaussian).

Supplementary Note 4: Time-resolved $\Delta\mathrm{Re}\chi^{(2)}$ spectra of the air/water interfaces

Supplementary Figure 4 shows the steady-state $\mathrm{Re}\chi^{(2)}$ spectra and time-resolved $\Delta\mathrm{Re}\chi^{(2)}$ spectra observed with selective excitation of the free OH at the delay times from –3.0 to 6.0 ps. These $\Delta\mathrm{Re}\chi^{(2)}$ and $\Delta\mathrm{Im}\chi^{(2)}$ (Figure 2 in the main text) were used for calculating the temporal change of the $|\chi^{(2)} + \Delta\chi^{(2)}|^2 - |\chi^{(2)}|^2$ signals shown in Figure 5 in the main text, which correspond to the homodyne TR-VSFG signals.

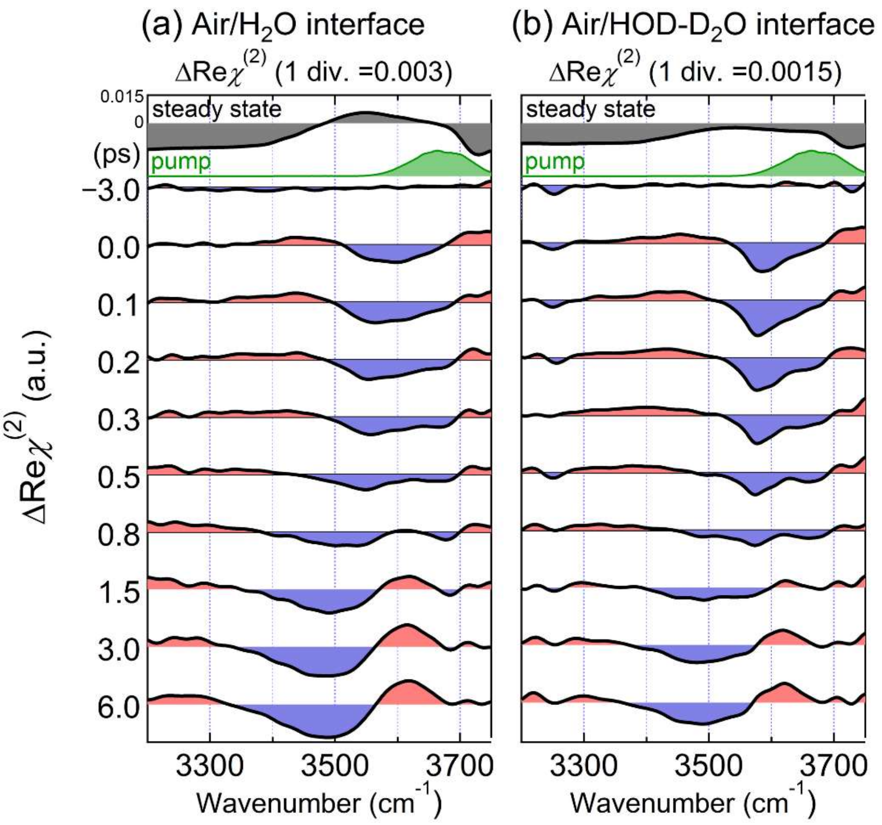

Supplementary Figure 4. **Time-resolved $\Delta\mathrm{Re}\chi^{(2)}$ spectra of the air/water interfaces.** Time-resolved $\Delta\mathrm{Re}\chi^{(2)}$ spectra obtained with selective excitation of the free OH, (a) neat H_2O and (b)

isotopically-diluted water ($H_2O/HOD/D_2O$ = 1/2/1) interfaces. Steady-state Re$\chi^{(2)}$ spectrum and the spectrum of the pump pulse are also shown at the top.

Supplementary Note 5: Influence of the perturbed free induction decay (PFID)

As shown in Figure 5 (b) in the main text, a small (~50 fs) difference is noticed between the rises of the transients in the free OH and HB OH regions of time-resolved ΔIm$\chi^{(2)}$ spectra. This difference is not due to the different temporal behavior of the signals arising from interfacial water but is attributable to the signal due to the perturbed free induction decay (PFID)[4] of the free OH. We describe the analysis that led us to this conclusion in the following.

In the time-resolved vibrational spectroscopic measurements, when the pump pulse arrives at the sample before the complete dephasing of the transition monitored, the pump pulse perturbs the free induction decay of the transition and a relevant signal appears at negative pump-probe time delays (ΔT), which is called PFID.[4] In particular, the spectral feature due to the PFID is often observed in femtosecond time-resolved vibrational spectra because the typical vibrational dephasing time is as long as a few picosecond. In the present time-resolved HD-VSFG measurement, the influence of PFID can be checked directly in the time-domain when the raw data taken in the frequency domain are transformed to the time domain by Fourier transformation to extract the heterodyned SFG signals.[5,6] The HD-VSFG signal transformed to the time domain (I (t)) is expressed with Eq. (S2. 1),[5] and a typical example at ΔT = −6.0 ps is shown in Supplementary Figure 5 (a):

$$I(t) = |E_{\mathrm{SFG}}(t)|^2 + |E_{\mathrm{LO}}(t)|^2 + E_{\mathrm{SFG}}(t)E_{\mathrm{LO}}(t)^* + E_{\mathrm{SFG}}(t)^*E_{\mathrm{LO}}(t). \quad \text{(S2. 1)}$$

Here, E_{SFG} (t) and E_{LO} (t) are the electric fields of the SFG signal from the sample and local oscillator (LO) in the time domain, respectively. As seen in Supplementary Figure 5 (a), the first two homodyne terms provide the signal at around the origin of the time axis (t = 0), whereas the third and fourth terms, which correspond to the heterodyned terms, appear at around t = −3.2 ps and 3.2 ps, which correspond to the delay between E_{SFG} (t) and E_{LO} (t).

For clarifying the effect of the pump pulse on the free induction decay, we examined the ratio of the time-domain SFG electric field measured at ΔT and that at −6.0 ps (E_{SFG} (t, ΔT) / E_{SFG} (t, −6.0 ps)). Supplementary Figure 5 (b) shows the ratio E_{SFG} (t, ΔT) / E_{SFG} (t, −6.0 ps) at different ΔT, which were calculated for the time-domain signals around t = −3.2 ps that correspond to the third term (E_{SFG} (t) E_{LO} $(t)^*$) in Eq. (S2. 1). As shown, when the pump pulse arrives after the complete dephasing of the free OH (ΔT = −3.0 ps), the SFG electric field is identical to that at −6.0 ps and thus the amplitude ratio is entirely 1 in the whole t region. (Note that FWHM of the free OH band is 24 cm^{-1},[7,8] which corresponds to the dephasing time (T_2) of ~0.4 ps.) On the other hand, when the pump pulse arrives before the complete dephasing of the free OH (ΔT = −0.2 and −0.1 ps), the amplitude ratio starts deviating from the unity at the time when the pump pulse excites the free OH. (The corresponding time points are indicated by red and blue arrows in Supplementary Figure 5 (b).) This change of the SFG electric field in the time domain represents PFID. In the positive pump-probe time delays (ΔT = 0.0 and 0.05 ps), the SFG electric field deviates from the unity in the whole t range, reflecting the appearance of the transient signal due to the interfacial water. This time-domain analysis of the SFG electronic field clearly shows that the Im$\chi^{(2)}$ signals at negative pump-probe time delays are affected by PFID.

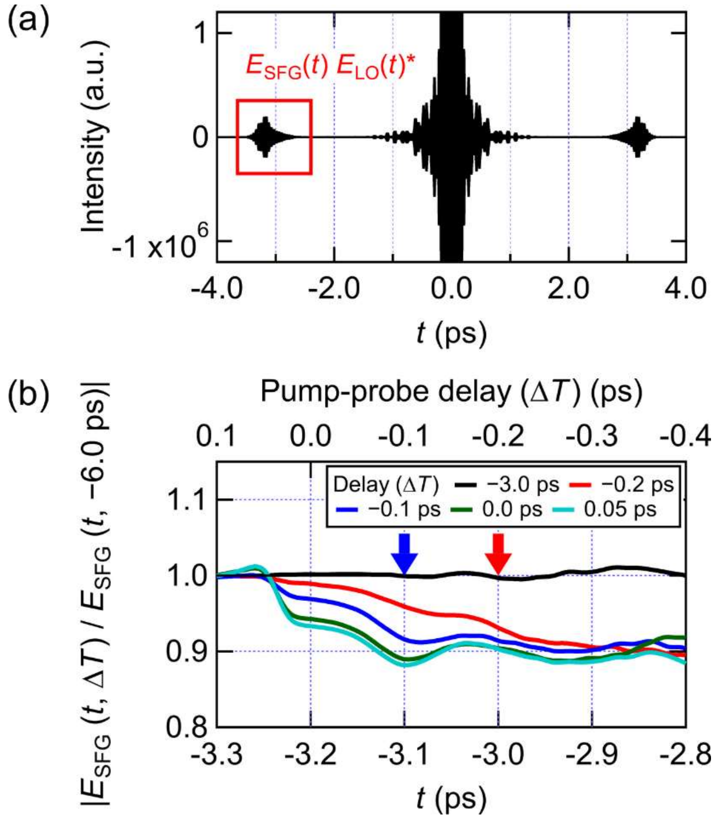

Supplementary Figure 5. **Perturbed free induction decay (PFID) recognized in the time-domain HD-VSFG signals.** (a) HD-VSFG signal in the time domain at $\Delta T = -6.0$ ps. The signal corresponding to the third term of Eq. (S2. 1) ($E_{\mathrm{SFG}}(t)\, E_{\mathrm{LO}}(t)^*$) is indicated with a red square. (b) Amplitude ratios of the time-domain SFG electric field at different pump-probe time delays (ΔT) with respect to that at −6.0 ps, i.e., $E_{\mathrm{SFG}}(t, \Delta T)$ / $E_{\mathrm{SFG}}(t, -6.0$ ps$)$. Red and blue arrows represent the arrival time points of the pump pulse in the measurements at $\Delta T = -0.2$ ps and −0.1 ps, respectively.

Based on the analysis described above, we sketch the influence of PFID on the temporal profile of the transient signal in Supplementary Figure 6. Because the pump pulse is resonant with the free OH, the PFID mainly affects the signal in the free OH region while giving rise to a small oscillating feature in the off-resonant region.[4] This influence of PFID is the largest when

the pump pulse comes just after the probe (i.e., at small negative ΔT), and it decreases as the pump-probe delay ΔT becomes more negative (as the amplitude of the free induction decay at the arrival time of the pump pulse becomes smaller.) Therefore, the temporal profile of the transient signal in the free OH region consists of (1) the signal due to PFID which decays toward the negative time with T_2 (0.4 ps) and (2) the true transient signal that appears with excitation of the free OH and decays in the positive time region with T_1 (0.9 ps) (Supplementary Figure 6 (a)). In contrast, the temporal profile of the transient in the HB OH region is considered to solely consist of the true transient signal that appears with excitation of the free OH and decays in the positive time with T_1 (0.9 ps) (Supplementary Figure 6 (b)). Consequently, after convolution with the 200-fs time resolution, the contribution of PFID gives rise to a small difference in the apparent rise between the transients in the free OH and HB OH regions. It makes the appearance of the former look faster than the latter by 50 fs.

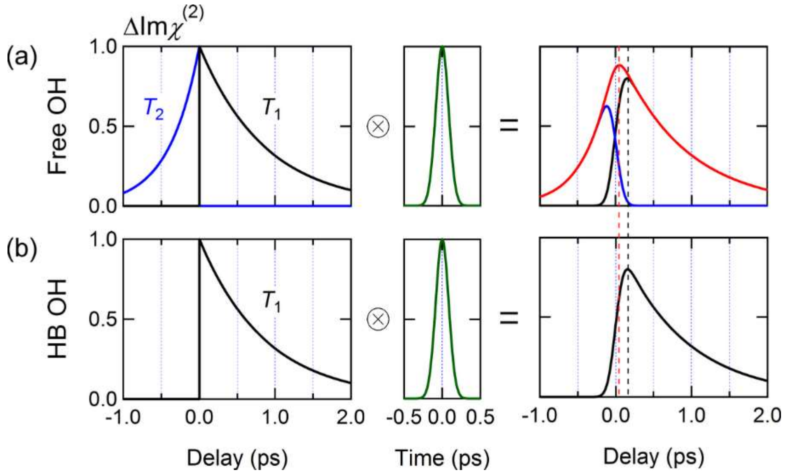

Supplementary Figure 6. **Schematic temporal profiles of the transients observed with selective excitation of the free OH.** (a) Temporal profile in the free OH region, which increases with T_2 (0.4 ps, blue, due to PFID) and decays with T_1 (0.9 ps, black). (b) Temporal profile in the HB OH region, which decays with T_1 (0.9 ps, black). For each case, the left panel depicts the

temporal response of the system itself, ⊗ denotes the convolution with 200-fs Gauss function, and the right panel shows the actual temporal profile measured with the apparatus. Dotted lines in the right panels represent the peak of the temporal profiles in the free OH region (red) and the HB OH region (black).

Supplementary References

1. Yamaguchi, S. & Hamaguchi, H.-o. Femtosecond ultraviolet-visible absorption study of all-*trans*→13-*cis*·9-*cis* photoisomerization of retinal. *J. Chem. Phys.* **109**, 1397-1408 (1998).

2. Matsuzaki, K., Kusaka, R., Nihonyanagi, S., Yamaguchi, S., Nagata, T. & Tahara, T. Partially Hydrated Electrons at the Air/Water Interface Observed by UV-Excited Time-Resolved Heterodyne-Detected Vibrational Sum Frequency Generation Spectroscopy. *J. Am. Chem. Soc.* **138**, 7551-7557 (2016).

3. Ahmed, M., Inoue, K., Nihonyanagi, S. & Tahara, T. Hidden Isolated OH at the Charged Hydrophobic Interface Revealed by Two-Dimensional Heterodyne-Detected VSFG Spectroscopy. *Angew. Chem. Int. Ed.* **59**, 9498-9505 (2020).

4. Hamm, P. Coherent effects in femtosecond infrared spectroscopy. *Chem. Phys.* **200**, 415-429 (1995).

5. Yamaguchi, S. & Tahara, T. Heterodyne-detected electronic sum frequency generation: "Up" versus "down" alignment of interfacial molecules. *J. Chem. Phys.* **129**, 101102 (2008).

6. Nihonyanagi, S., Yamaguchi, S. & Tahara, T. Direct evidence for orientational flip-flop of water molecules at charged interfaces: A heterodyne-detected vibrational sum frequency generation study. *J. Chem. Phys.* **130**, 204704 (2009).

7. Wei, X. & Shen, Y. R. Motional Effect in Surface Sum-Frequency Vibrational Spectroscopy. *Phys. Rev. Lett.* **86**, 4799-4802 (2001).

8. Yamaguchi, S. Development of single-channel heterodyne-detected sum frequency generation spectroscopy and its application to the water/vapor interface. *J. Chem. Phys.* **143**, 034202 (2015).
